# An Assessment of Inter-Observer Agreement in Water Source Classification and Sanitary Risk Observations

**DOI:** 10.1007/s12403-019-00339-3

**Published:** 2019-12-24

**Authors:** Joseph Okotto-Okotto, Peggy Wanza, Emmah Kwoba, Weiyu Yu, Mawuli Dzodzomenyo, S. M. Thumbi, Diogo Gomes da Silva, Jim A. Wright

**Affiliations:** 1 Victoria Institute for Research on Environment and Development (VIRED) International, off Nairobi Road, Rabour, P.O. Box 6423-40103, Kisumu, Kenya; 2 Centre for Global Health Research, Kenya Medical Research Institute, P.O. Box 1578-40100, Kisumu, Kenya; 3 School of Geography and Environmental Science, University of Southampton, Building 44, Highfield, Southampton, SO17 1BJ UK; 4 Ghana School of Public Health, University of Ghana, P.O. Box LG 13, Legon, Accra, Ghana; 5 Paul G Allen School for Global Animal Health, Washington State University, Pullman, WA 99164- 7090 USA; 6 School of Environment and Technology, University of Brighton, Cockcroft Building, Lewes Road, Brighton, BN2 4GJ UK

**Keywords:** Water safety, Rainwater, Inter-observer agreement, Sanitary inspection, Sustainable development goals

## Abstract

Sanitary risk inspection, an observation protocol for identifying contamination hazards around water sources, is promoted for managing rural water supply safety. However, it is unclear how far different observers consistently identify contamination hazards and consistently classify water source types using standard typologies. This study aimed to quantify inter-observer agreement in hazard identification and classification of rural water sources. Six observers separately visited 146 domestic water sources in Siaya County, Kenya, in wet and dry seasons. Each observer independently classified the source type and conducted a sanitary risk inspection using a standard protocol. Water source types assigned by an experienced observer were cross-tabulated against those of his colleagues, as were contamination hazards identified, and inter-observer agreement measures calculated. Agreement between hazards observed by the most experienced observer versus his colleagues was significant but low (intra-class correlation = 0.49), with inexperienced observers detecting fewer hazards. Inter-observer agreement in classifying water sources was strong (Cohen’s kappa = 0.84). However, some source types were frequently misclassified, such as sources adapted to cope with water insecurity (e.g. tanks drawing on both piped and rainwater). Observers with limited training and experience thus struggle to consistently identify hazards using existing protocols, suggesting observation protocols require revision and their implementation should be supported by comprehensive training. Findings also indicate that field survey teams struggle to differentiate some water source types based on a standard water source classification, particularly sources adapted to cope with water insecurity. These findings demonstrate uncertainties underpinning international monitoring and analyses of safe water access via household surveys.

**Electronic supplementary material:**

The online version of this article (10.1007/s12403-019-00339-3) contains supplementary material, which is available to authorized users.

## Background

Target 6.1 of the Sustainable Development Goal (SDG) aims to ‘By 2030, achieve universal and equitable access to safe and affordable drinking water for all’ (United Nations 2019). To deliver this target, the World Health Organization has promoted water safety plans as a tool for rural water supply managers to ensure the safety of such supplies (Rickert et al. 2014). In remote and resource-poor settings, however, microbiological testing is often unavailable given its cost, lack of consumables or distance to laboratory infra-structure and skilled staff (Wright et al. 2014), with far less testing being completed on non-piped than piped supplies in sub-Saharan Africa (Kumpel et al. 2016). Where microbiological testing does take place, supply managers require methods for identifying the hazards responsible for the microbiological contamination identified through water testing so that these can be remediated. For this reason, the World Health Organization (WHO) has promoted the use of structured observation protocols for identifying faecal contamination hazards at and surrounding rural water sources (World Health Organization 1997). These protocols, often referred to as sanitary risk inspections, identify hazards such as problems with the structural integrity of source protection measures (e.g. blocked drainage channels or broken fencing around protected wells) and contamination sources in the surrounding environment (e.g. pit latrines or livestock immediately upstream of a spring). As well as being promoted as a tool for water supply managers, sanitary risk inspection has also been used in national water source surveys, such as the Rapid Assessment of Drinking-Water Quality (RADWQ) survey series (World Health Organization and UNICEF 2012).

Although sanitary risk inspection has been promoted for over two decades and has been widely used in many settings (Ercumen et al. 2017; Howard et al. 2003; Luby et al. 2008), it is unclear how reliably different surveyors can identify a given set of hazards at or surrounding a given set of water sources using these protocols. Reliability refers to the repeatability or consistency of measurements (Heale and Twycross 2015) and may vary both over time and between observers. Consistency in repeated measurements based on the same protocol is often referred to as stability, whilst consistency in measurements made by different observers using the same protocol is referred to as equivalence (Heale and Twycross 2015). In public health, inter-observer agreement studies are commonly used to assess whether observations or measurements can reliably be made by community-based healthcare professionals (Laar et al. 2018; Triasih et al. 2015) rather than specialists. However, studies of inter-observer agreement are less common in low and middle income countries (Bolarinwa 2015) and environmental management. If sanitary risk inspection protocols are to form a robust basis for water source remediation or comparing the relative safety of sources in different areas via water source surveys, then observations need to be consistent across observers. We recently conducted a small-scale study of inter-observer agreement of sanitary risk observations at groundwater sources in Greater Accra, Ghana, finding high agreement between two observers (Yentumi et al. 2018). To avoid personal safety risks from lone working, both observers visited water sources in Greater Accra at the same time, though they observed and recorded hazards separately. However, because both observers visited sources simultaneously, the behaviour of one observer (e.g. in searching behind buildings for hazards) could have influenced the behaviour of the second observer. Other than this study, to our knowledge there have been no other published studies of this issue.

Whilst domestic livestock play a valuable role in rural livelihoods, contributing to nutrition, financial income and food crop production via draught power and manure (Randolph et al. 2007), livestock ownership can also contribute to health risks, as several common diarrhoeal pathogens (e.g. *Salmonella* and *Campylobacter* spp., *Cryptosporidium parvum*, *E. coli* O157 and *Giardia duodenalis*) can be harboured by animals (Du Four et al. 2012). In Kenya, the Global Enteric MultiCenter Study of diarrhoeal disease found *Cryptosporidium* spp. to be the second leading pathogen associated with child diarrhoea (Kotloff et al. 2013). A systematic review found 69% of studies assessing the relationship between domestic animal husbandry and human diarrhoeal disease reported a significant positive association, and this increased to 95% in studies assessing pathogen-specific diarrhoea (Zambrano et al. 2014). This indicates that domestic livestock may be an important source of diarrhoeal pathogens. Run-off of animal faeces into sources and/or sharing of water sources by livestock and people has been identified as one of several potential transmission routes, alongside a need for more robust field observation protocols for such hazards (Penakalapati et al. 2017).

Alongside this, concerns have also been expressed over the potential for misclassification of water source types when identifying the primary source used by households (Bartram et al. 2014). A core question and set of response categories has been developed to record a household’s main drinking-water source (WHO/UNICEF 2006) and subsequently revised (UNICEF 2018). This is incorporated into household surveys such as Demographic and Health Surveys (DHS) and used to support international monitoring of progress towards SDG target 6.1 (United Nations 2019). However, the extent of uncertainty arising from ambiguity in water source classification remains unclear as, to our knowledge, there are no previous studies of such classification ambiguity.

The objectives of this study are therefore to assess inter-observer agreement in sanitary risk inspections and thereby strengthen the field protocols used to manage the safety of rural water supplies. A subsidiary objective is to quantify uncertainty in the classification of water source types. In doing so, we seek to build on our earlier study (Yentumi et al. 2018), addressing some of its limitations and expanding the study design to include observations by more than two observers and other rural water source types, notably rainwater harvesting systems. As a second subsidiary objective, we also seek to assess the robustness of observational evidence for contact between water sources and livestock and thereby the implications for diarrhoeal disease control.

## Methods

### Study Site

Fieldwork took place in ten villages in Siaya County, Kenya, a rural site on the shores of Lake Victoria, which hosts a Health and Demographic Surveillance System (Odhiambo et al. 2012) and where residents participate in several ongoing studies of livestock and human health (Thumbi et al. 2015). These studies suggested 43% of households collected domestic water from wells, 32% used rainwater or seasonal streams, whilst most of the remaining households relied on surface water from dams, pans or the lake (Thumbi et al. 2015). Most households (82%) reported having at least one outdoor latrine.

### Protocol Development and Field Team Recruitment and Training

Six observers participated in this exercise and were deliberately chosen to have varying levels of prior experience and education. The ‘gold standard’ observer (Joseph Okotto-Okotto, JOO; Observer A) had over 20 years’ experience of sanitary risk observation, publishing several papers on this topic (Okotto-Okotto et al. 2015; Wright et al. 2013) and managing multiple rural water supply projects. A second (Observer E) also had previous experience of sanitary risk observation and some tertiary education and together with two recent graduates (Observers B and F) were recruited to typify survey team members who might support a regional or national water point mapping exercise. The remaining two (Observers C and D) had a further education qualification and only basic secondary education respectively, and were recruited to typify community-based water user committee members, who might be tasked with ongoing water safety management of rural supplies.

JOO led 4 days’ training of the other five observers, including reviewing the implementation of the water source classification and sanitary risk protocols in detail, estimating distances via pacing and inspecting rainwater roof catchment areas. The survey team then piloted all tools in villages outside the study sites, initially surveying sources as a group and then individually, recording findings via CommCare. A 2-day refresher training session was held before the second fieldwork period.

Following initial piloting, sanitary risk inspection protocols were adapted from those promoted by WHO (World Health Organization 1997). Adaptations involved checking for the presence of water system components (e.g. filter boxes on rainwater systems; parapets surrounding wells), additional observations concerning livestock hazards (e.g. footprints or animal faeces at a source) and additional observations of a hazard’s underlying causes (e.g. branches overhanging a roof catchment for rainwater harvesting, leading to bird droppings). Protocols were selected based on six source types: springs, surface waters, unprotected wells, protected wells, boreholes and rainwater harvesting systems (see Table 1 for example). Following piloting, the pre-2018 JMP core question concerning the main source of drinking-water (WHO/UNICEF 2006) was adapted to include an additional response category for water kiosks. Following a team review and follow-up site visits after wet season fieldwork, it emerged that some households were fetching water from broken pipes. Others had adapted their water supplies to cope with intermittent supplies by storing piped and rainwater in the same tank. Specific response categories were introduced for such sources in the subsequent visit.

**Table 1:** Summary table of adapted sanitary risk inspection checklist for rainwater harvesting systems

Component	Sanitary risk items	Responses
Roof catchment	Risk present	(1) Yes; (2) no
	Type of risk	(1) Birds/bird droppings; (2) plants/leaves; (3) overhanging branches; (4) other (specify)
Guttering channel	Dirty gutters	(1) Yes;( 2) no; (3) don’t know (cannot see)
	Moveable inlet pipe	(1) Yes; (2) no; (3) don’t know (cannot see)
Tank inlet	Filter box	(1) Yes; (2) no; (3) don’t know (cannot see)
	Inside tank inlet	(1) Fine gravel; (2) coarse stones/gravel; (3) debris/leaves/dirt; (4) sieve; (5) other (specify)
Tank cover	Improper cover	(1) Yes; (2) no; (3) don’t know (cannot see)
Storage tank	Defect (e.g. cracks) in the walls / on the top	(1) Yes; (2) no
	Depression on the top	(1) Yes; (2) no
Tap	Tap present	(1) Yes; (2) no; (3) don’t know (cannot see)
	Tap defective/leaking	(1) Yes; (2) no
Concrete floor	Concrete floor present	(1) Yes; (2) no
	Risk present	(1) Cracked; (2) broken; (3) dirty
Drainage	Inadequate drainage	(1) Yes; (2) no
Water fetching method	Bucket/container for fetching water	(1) Yes; (2) no
	Bucket/container left on the ground	(1) Yes; (2) no
	Dirty bucket/container	(1) Yes; (2) no

### Sample Design and Water Source Selection

To estimate the minimum required sample size for our study, we used the published method for approximating the variance of the estimated limits of agreement (Bland and Altman 1999), and the standard deviation of differences between percentage sanitary risk scores recorded by two observers of wells and boreholes in Greater Accra, Ghana (Yentumi et al. 2018). We estimated that observations of at least 92 water sources would give 95% confidence limits of 19.91% for the limits of agreement.

Chosen water sources were drawn from those used by 234 households participating in the OneHealthWater study (https://www.onehealthwater.org/). After seeking their informed consent to participate in the study, households were asked to identify the main drinking-water source that they used in an initial visit. These sources were visited by the survey team between 9th April 2018 and 4th June 2018, the season of long rains. Households were revisited in the dry season and asked to identify the source used to obtain drinking-water stored in the home at the time of the visit. These sources were then visited between 21st November 2018 and 2nd March 2019, alongside those previously reported as used by households in the first visit.

### Fieldwork

During wet season fieldwork, the six observers visited each of these sources independently at different times to reduce the potential for collusion or one observer’s behaviour influencing a second observer. In the subsequent visit, only five observers (A–C; E–F) were available to conduct fieldwork. Logistical difficulties in organising visits in this rural area sometimes led to a lag of several days between successive visits to the same source, particularly in the wet season. Each observer first identified the appropriate source class based on the adapted version of the JMP’s standard classification (WHO/UNICEF 2006) and an accompanying pictorial guide. Observer B additionally collected a water sample and took in-situ measurements of turbidity and electro-conductivity using a Hanna Instruments HI 93,703 and a COND3110 portable meter respectively. If the source type was rainwater, a well, borehole, spring or surface water, each observer undertook a sanitary risk inspection to identify contamination hazards at or surrounding each source, based on the observation protocol for that source type (see the supplementary video online at https://generic.wordpress.soton.ac.uk/onehealthwater/work-overview/sanitary-risk-observation/). Piped water sources and water vended from kiosks were thus excluded from sanitary risk inspections. Where a hazard such as a latrine was identified close to a source, the observers estimated the distance to the hazard by pacing and using a self-determined pace factor to convert paces to distances. All observations were recorded via the CommCare cell phone-based data collection system (Dimagi Inc 2019). Unless the field team was explicitly asked by bystanders, no feedback was provided on the hazards present during the visit.

### Analysis

To assess ambiguity in classification of water source types, we cross-tabulated the source type assigned by Observer A against those assigned by the other five observers.

In the absence of expert hydrogeological advice, we assumed that 30 m constituted a safe horizontal separation distance between contamination hazards (e.g. pit latrines) and wells, springs and boreholes, since this has previously been used as a conservative threshold for safe lateral separation between source and hazard (Howard et al. 2003). We then calculated the kappa index of agreement (McHugh 2012) separately for each hazard observation, based on records from all six observers. We graphically compared the distance to nearest latrine estimated by Observer A against those estimated by the remaining five observers, calculating Lin’s concordance correlation coefficient and related statistics (Bradley and Blackwood 1989) for these estimates using the Stata version 15.0 *concord* and *batplot* utilities.

For each source and observer, we calculated a percentage sanitary risk score as the number of hazards present as a proportion of those observed, following common practice in analysing such data (Howard et al. 2003; Misati et al. 2017; Okotto-Okotto et al. 2015). We again computed Bland and Altman limits of agreement and related statistics for Observer A’s records against those of each of the remaining five observers. We also calculated absolute intra-class correlation coefficients for the sanitary risk scores from all six surveyors’ observations, based on a two-way random effects model (Koo and Li 2016), separately for each source type and for all sources combined.

To explore potential influences on disagreement between observers, we fitted linear regression models to predict the absolute difference between Observer A’s risk scores and those of each of his colleague. Alongside source type and observer, we examined indicators of observer fatigue (time of day and week when sources were surveyed and sequential order of source visits made); possible impact of protocol deviations (the absolute lag in days between Observer A’s visit and that of his colleague and whether one of a pair of source surveys was the first to be made) and changes in environmental conditions. We measured the latter as the absolute difference in daily rainfall on dates when the two surveys were made, obtaining rainfall estimates from the Climate Hazards Group Infra-Red Precipitation with Station (CHIRPS) gridded data product, which is based on satellite imagery and in-situ measurements (Funk et al. 2014). Because of unseasonal rains, this hydro-meteorological classification identified part of the Visit 2 fieldwork period as being in the wet season, so we hereafter refer to this as ‘partially dry’. We generated locally weighted smoothed scatterplots of continuous variables, subsequently fitting univariate (unadjusted) and then multivariate (adjusted) linear regressions of variables significant at the 99% level in univariate models.

## Results

### Summary of Sources Surveyed

Table 2 (below) shows the types of water sources surveyed in each season, classified by the most experienced observer who visited each source. In both seasons, the most widespread source type surveyed was rainwater, followed by piped water. When new response categories were introduced for ‘hybrid’ sources in the second visit (e.g. systems adapted to draw on both piped and rainwater to cope with intermittent supply), these comprised 11.6% of all sources used.

**Table 2:** Types of drinking-water source surveyed in wet and partially dry seasons, classified according to the most experienced observer visiting each source (^1^Source types used in second visit only)

Source type	Water sources surveyed by JOO in both visits		All water sources surveyed	
	Visit 1 (wet season)	Visit 2 (partially dry season)	Visit 1 (wet season)	Visit 2 (partially dry season)
Piped to yard/plot	14 (18.4%)	11 (14.5%)	17 (17.7%)	17 (11.6%)
Standpipe/public tap	1 (1.3%)	3 (3.9%)	4 (4.2%)	5 (3.4%)
Tubewell/borehole	3 (3.9%)	3 (3.9%)	4 (4.2%)	4 (2.7%)
Protected well	9 (11.8%)	10 (13.2%)	11 (11.5%)	17 (11.6%)
Unprotected well	3 (3.9%)	0 (0.0%)	3 (3.1%)	0 (0.0%)
Unprotected spring	0 (0.0%)	0 (0.0%)	0 (0.0%)	1 (0.7%)
Rainwater	19 (25.0%)	15 (19.7%)	26 (27.1%)	45 (30.8%)
River/stream	4 (5.3%)	4 (5.3%)	6 (6.3%)	6 (4.1%)
Lake/dam/pan	17 (22.4%)	17 (22.4%)	19 (19.8%)	25 (17.1%)
Kiosk	6 (7.9%)	5 (6.6%)	6 (6.3%)	7 (4.8%)
Rainwater-piped hybrid^1^	–	6 (7.9%)	–	12 (8.2%)
Burst pipe^1^	–	0 (0.0%)	–	2 (1.4%)
Spring/well-dam/pan hybrid^1^	–	2 (2.6%)	–	5 (3.4%)
Total	76	76	96	146

**Fig. 1:**
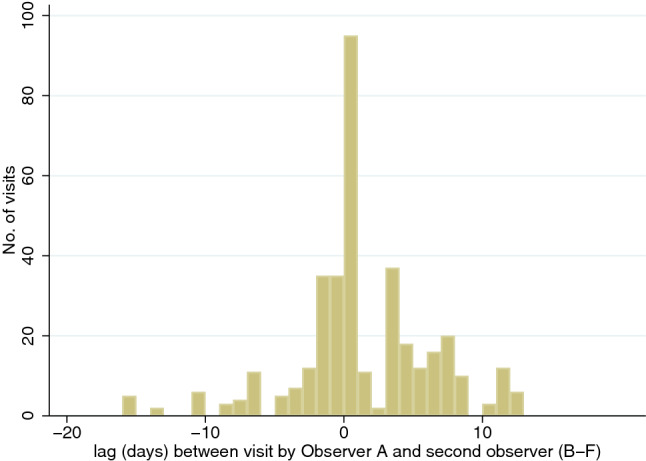
Number of days between visits to the same water sources by the most experienced observer (Observer A) versus five other field team members (Observers B to F) in the wet season

**Fig. 2:**
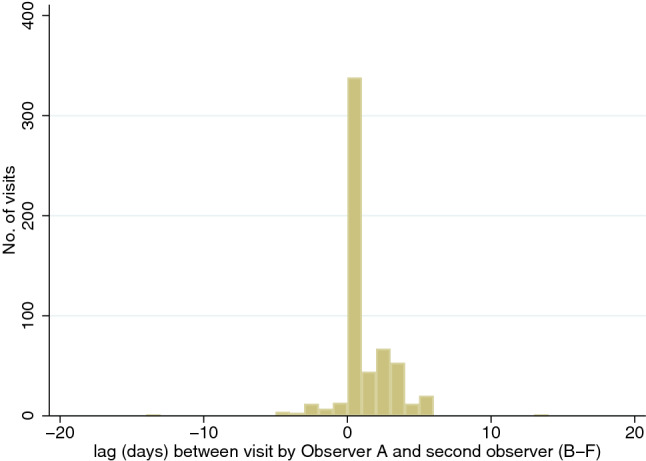
Number of days between visits to the same water sources by the most experienced observer (Observer A) versus five other field team members (Observers B to F) in the partially dry season (excludes 4 lags greater than a month)

In the wet season, because of logistical issues, 61 sources were surveyed independently by all six observers, 17 by five observers, seven by four and seven by three observers, two by two observers and two by one observer. 80 sources were surveyed by the most experienced observer. In the partially dry season, 141 sources were visited by all five observers, whilst five were visited by four observers. All sources were visited by the most experienced observer in the partially dry season. Figures 1, 2 show the lag in days between Observer A’s survey visits and those of the other observers. In the wet season, the greatest time gaps between source surveys were Observer A’s visit occurring 16 days before that of a colleague and 13 days after (median 0 days; inter-quartile range 5 days). In the partially dry season, in four instances the lag was greater than a month, but otherwise Observer A’s visits occurred a maximum of 14 days prior to his colleagues and 14 days after (median 0 days; inter-quartile range 2 days).

Figures 1, 2 shows the lag in days between the most experienced observer’s visit to each water source and those of the other team members.

### Inter-Observer Agreement in Water Source Classification

Table 3 shows the classification of water source types in the wet season by the most experienced observer, JOO, versus how the other five observers independently classified water source types. 86.3% of the other team members’ identified source types agreed with JOO (Kappa index of agreement = 0.835, 95% confidence intervals (CI) 0.827–0.899). Examining discrepancies, relative to the most experienced observer, the other observers often classified piped supply points as public standpipes, rather than being piped to household premises. There were also some discrepancies between the most experienced observer and the rest of the team over the classification of boreholes, unprotected springs, protected and unprotected wells. Kiosks were also frequently confused with piped water, reflecting the practice of purchasing or temporarily using water from neighbours’ taps.

**Table 3:** Cross-tabulation of water source type classification made by the most experienced observer (JOO—rows) versus five less experienced observers (columns) for 80 water sources during the first visit

	Piped to premises	Standpipe	Borehole	prot. well	unprot well	unprot spring	Rainwater	Surface water	Kiosk	Total
Piped to premises	**49**	8	0	0	0	0	0	0	3	60
Standpipe	4	**5**	0	0	0	0	0	0	0	9
Borehole	0	0	**16**	3	0	0	0	0	1	20
Protected well	0	0	1	**38**	0	0	0	0	0	39
Unprotected well	0	0	0	3	**9**	2	0	1	0	15
Unprotected spring	0	0	0	0	0	**0**	0	0	0	0
Rainwater	6	0	0	0	0	0	**85**	0	0	91
Surface water	0	0	0	0	0	0	0	**105**	0	105
Kiosk	1	16	0	0	0	0	0	0	**10**	27
Total	60	29	17	44	9	2	85	106	14	366
% agreement with experienced observer	81.7%	17.2%	94.1%	86.4%	100.0%	0.0%	100.0%	99.1%	71.4%	

The most experienced observer classified six systems as rainwater harvesting, whilst the other observers considered them piped systems. On follow-up review, these ambiguously classified water systems proved to be ‘hybrid’ systems, with tanks capable of storing both piped and rainwater, as an adaptation to the intermittency of both piped supplies and rainwater. Similarly, JOO classified one source as an unprotected well, whereas the rest of the team classified this as a water pan. Follow-up fieldwork revealed this source had again been modified by consumers to cope with water scarcity, with a retaining wall built to hold water when it overflowed. Retrospective inspection of problematic sources identified eight such hybrid rainwater-piped sources and two hybrid well/spring-surface water sources. These sources had been misclassified on 12 (31%) of 39 survey visits by the five less experienced observers, compared to a misclassification rate of 14% (46) for visits to the remaining sources.

Classification patterns in the subsequent visit to sources (Online Resource 1) were similar (kappa index of agreement 0.849, CI 0.846–0.901), with the introduction of additional source types for burst pipes, and ‘hybrid’ sources having minimal effect on inter-observer agreement. Burst pipes were accidental or deliberate breakages in the piped distribution system, from which households could collect water without payment.

### Reliability of Individual Hazard Observations

**Fig. 3:**
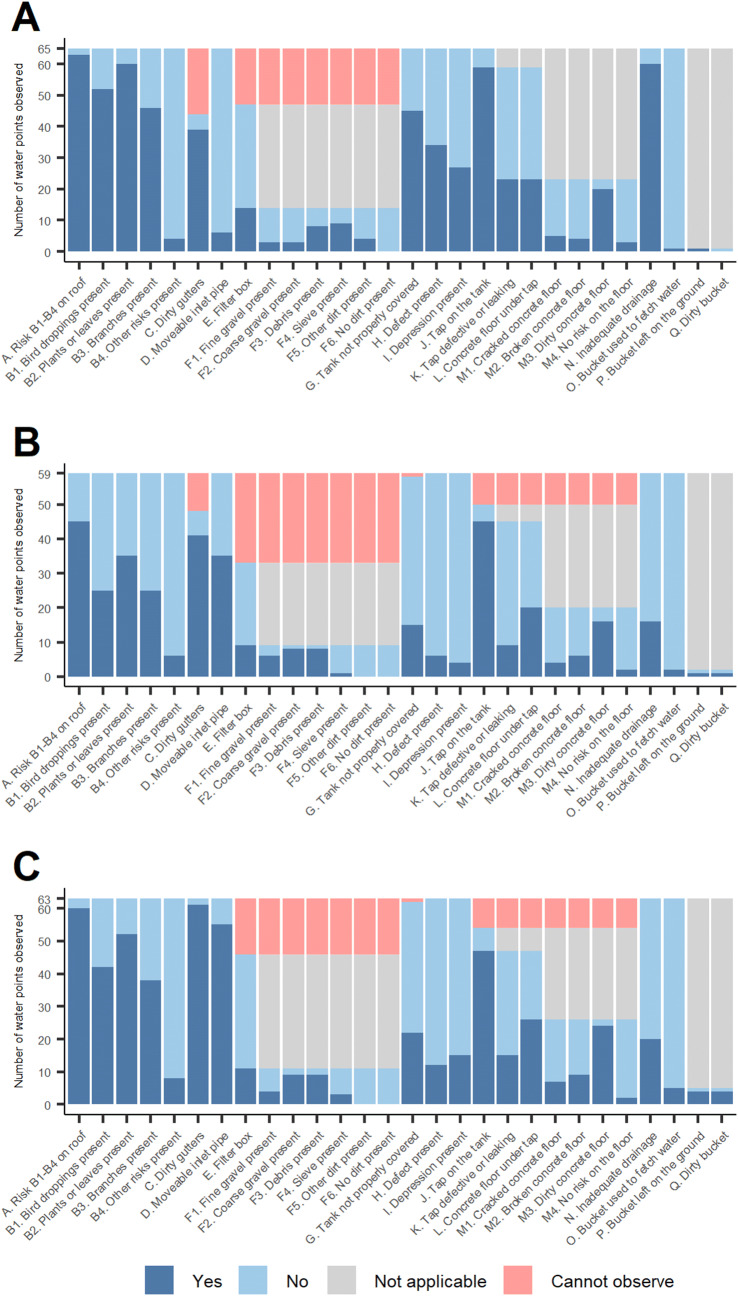
Hazard observations for 65 rainwater sources made by **a** Observer A (JOO, the most experienced observer); **b** Observer B; **c** Observer C

Figure 3 shows the hazard observations made at 65 rainwater sources for the most experienced observer, versus two of his colleagues. Roof catchments and gutters were identified as having the most widespread hazards by all three observers. In general, the most experienced observer detected more hazards than his colleagues, particularly in inspecting the integrity of the storage tank. For some rainwater systems, surveyors were unable to observe the state of guttering and presence or absence of filter boxes to prevent debris entering the tank. Online Resource 2 contains the calculated kappa indices of agreement for all hazard observation items, for all water sources. For rainwater sources, the greatest consistency in hazard observations was over the absence of a filter box and the state of any bucket used, with no significant agreement over roof catchment hazards and minimal but significant agreement over other hazard observations.

**Fig. 4:**
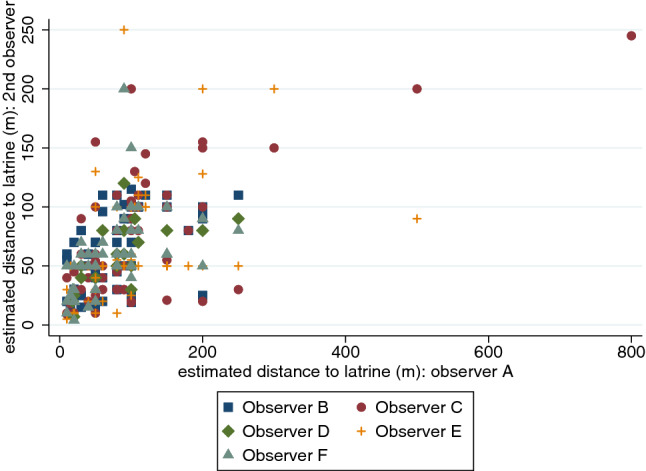
Distance to nearest latrine, measured through pacing by the most experienced observer (Observer A – JOO) versus the other five observers

Figure 4 shows the inter-observer variation in the distance estimated by pacing from all source types to the nearest latrine. Estimated distances were moderately correlated with that of the most experienced observer (*R* = 0.36 to 0.65).

### Reliability of Sanitary Risk Scores

**Fig. 5:**
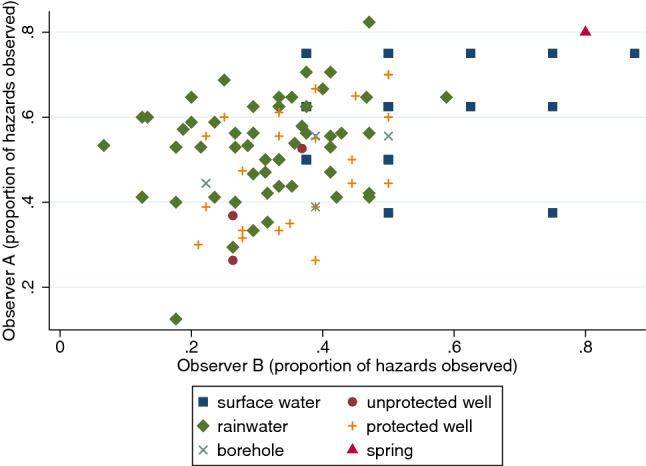
Sanitary risk scores for 146 water sources in wet and partially dry seasons showing Observer A (JOO, the most experienced observer) versus Observer B

**Fig. 6:**
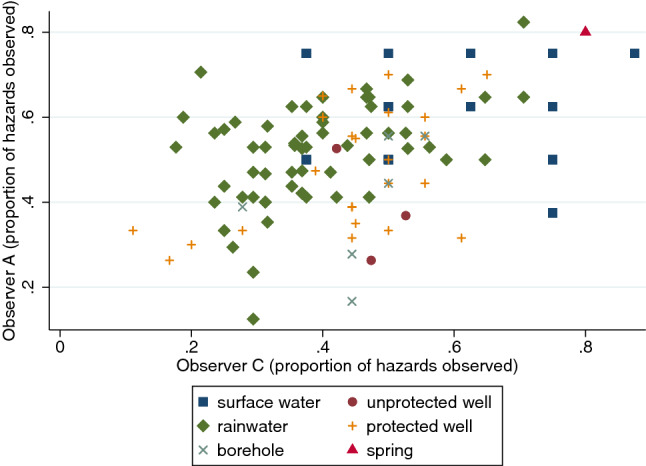
Sanitary risk scores for 146 water sources in wet and partially dry seasons showing Observer A (JOO, the most experienced observer) versus Observer C

Figures 5, 6 (below) show the sanitary risk scores for the most experienced observer (Observer A; JOO) versus two of his colleagues from both wet and partially dry seasons. Following a similar pattern to the scores for Observers D–F (not shown), Observer B’s scores were generally lower than Observer A’s indicating (s)he had observed fewer contamination hazards (Fig. 5). Observer C’s scores were on average closer to Observer A’s scores (Fig. 6). The graphics for Observer B and C suggest low agreement with Observer A’s scores within some source types, particularly rainwater.

**Fig. 7:**
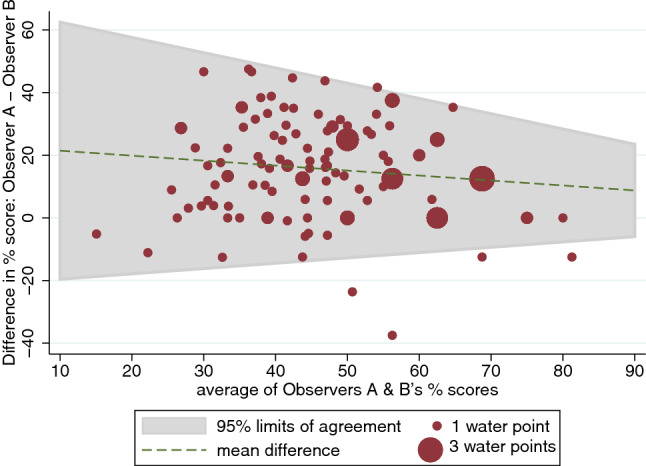
Bland and Altman plot for sanitary risk scores calculated for Observer A (JOO; the most experienced observer) versus Observer B

**Fig. 8:**
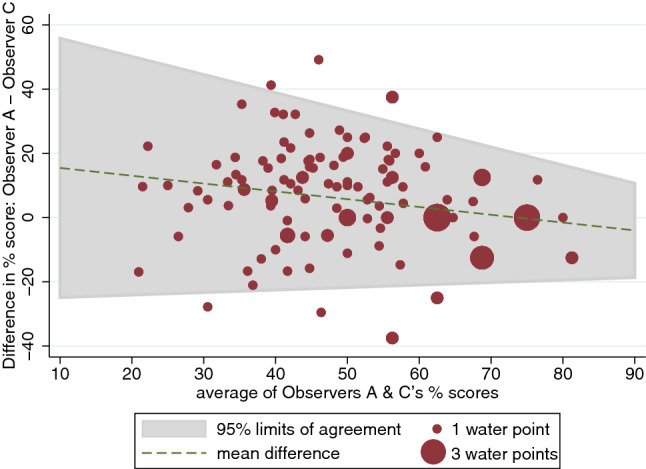
Bland and Altman plot for sanitary risk scores calculated for Observer A (JOO; the most experienced observer) versus Observer C

Figures 7, 8 show the Bland and Altman plot of the risk scores for all water source types combined, based on JOO’s observations versus those of Observers B and C. As shown by the differences in risk scores plotted on the y-axis, JOO’s risk scores were generally higher than those for the other two observers. This suggests that the more experienced observer, JOO, identified more hazards than these other two observers.

Table 4 summarises the findings of the Bland and Altman limits of agreement analysis for the five less experienced observers, relative to the most experienced observer. All different source types have been grouped together for this analysis. The average differences in percentage risk scores show that all observers recorded fewer hazards than JOO, with four of these averaging risk scores that were more than 10% lower. Observer C’s risk scores were on average closer to JOO’s scores. Overall, correlation between risk scores was generally large, but lower for Observer F.

**Table 4:** Bland and Altman statistics for sanitary risk observations made by four observers, relative to the ‘gold standard’ Observer A (JOO) (***Indicates significance at alpha = 0.001)

	Versus Observer B	Versus Observer C	Versus Observer D	Versus Observer E	Versus Observer F
No. of water source visits	139	146	53	147	152
Lin’s concordance correlation coefficient (95% confidence intervals)	0.320 (0.22–0.42)	0.515 (0.40–0.63)	0.372 (0.20–0.55)	0.435 (0.33–0.54)	0.230 (0.15–0.31)
Pearson’s r	0.496	0.553	0.504	0.570	0.441
Bias correction factor	0.645	0.931	0.738	0.764	0.521
Average difference in % risk scores (95% limits of agreement)	15.4% (− 14.0% to 44.8%)	5.2% (− 24.4 to 34.8)	12.9% (− 20.5% to 46.4%)	11.9% (− 18.2% to 42.1%)	21.7% (− 15.0% to 58.5%)
Correlation between difference in risk scores and mean	− 0.14	− 0.22	− 0.32	− 0.29	− 0.39
Bradley-Blackwood F test	75.5 (***)	12.9 (***)	19.4 (***)	54.4 (***)	132.8 (***)

Table 5 shows the absolute intra-class correlation coefficients for the most frequently surveyed water source types, based on observations by the five observers participating in both wet and partially dry season fieldwork. ICCs for all source types were significant, but indicated low to moderate agreement. Agreement was poorest for rainwater, poor for surface water and approached moderate agreement for protected wells. The ICC was somewhat higher for all sources combined, reflecting some consistency across observers in the average percentage of hazards for each source type.

**Table 5:** Intra-class correlation coefficients for sanitary risk scores based on five surveyors’ observations over two visits to water sources

Source type	No. of source visits	Intra-class correlation (95% confidence limits)
Surface water	47	0.24 (0.12 to 0.39)
Rainwater	51	0.12 (0.02 to 0.26)
Protected wells	17	0.44 (0.22 to 0.68)
All sources	129	0.49 (0.33 to 0.62)

Online Resource 3 shows unadjusted and adjusted regression model coefficients for the absolute difference between Observer A’s risk scores and those of his colleagues. In both adjusted and unadjusted models, absolute differences in scores were significantly higher for rainwater sources, higher for Observer F and lower for Observer C. The absolute difference in scores increased significantly over time, particularly during the latter half of dry season fieldwork.

## Discussion

To our knowledge, there have been no previous studies of ambiguity in water source classification and only one previous study of inter-observer agreement in sanitary risk observation, despite widespread use of sanitary risk protocols (Mushi et al. 2012; Parker et al. 2010; Snoad et al. 2017) and their promotion by WHO for several decades (World Health Organization 1997).

Our inter-observer agreement study indicates that where households adopt strategies to cope with water insecurity, their sources proved difficult to classify unambiguously using the standard typology used for international monitoring (WHO/UNICEF 2006). Such strategies included construction of ‘hybrid’ sources to cope with water insecurity, such as water tanks that stored both rainwater and piped water to cope with the sporadic nature of rainwater and frequent interruptions to piped water. Other coping strategies highlighted in a systematic review of household adaptations to supply disruptions (Majuru et al. 2016) also led to ambiguous source classification, including use of burst pipes and reliance on neighbours’ taps for drinking-water. Whilst there has been growing recognition of the need to expand household survey content to incorporate previously overlooked water insecurity dimensions (Jepson et al. 2018; Wutich et al. 2018), the implications of water insecurity for *water source* classification as opposed to *household* surveys have not been considered. Such source adaptations could constitute valuable means for coping with water insecurity worthy of further investigation and wider dissemination if effective, yet they are not captured by the response categories used in household surveys such as the DHS. Whilst strategies for coping with water insecurity such as borrowing water from neighbours are believed to be quite widespread (Wutich et al. 2018; Zug and Graefe 2014), they were not directly captured in the pre-2018 JMP core questions on household drinking-water access. The latest revision to the core questions has since addressed this through inclusion of a response category concerning neighbour’s tap (UNICEF 2018). Water sources such as broken pipelines are however not mapped onto the water ‘ladder’ used for international monitoring. Whilst inclusion of additional response classes (e.g. use of neighbour’s taps) can reduce classification ambiguity, nonetheless some level of ambiguity may be inherent to any classification system. Where source type data from both enumerators and field supervisors undertaking back-audits are available for large-scale surveys, inclusion of a cross-tabulation of these in a data quality report appendix would quantify such ambiguity.

Furthermore, some source categories are often confused in the field. Most notably, these include the distinction between protected and unprotected wells; mechanised wells and boreholes and whether piped water is located on premises or is a public standpipe (see Table 3). Where well protection measures such as concrete aprons had fallen into disrepair, this made distinguishing protected versus unprotected wells particularly challenging. Unprotected wells constitute an unimproved source, the second lowest rung on the JMP’s ladder, whilst protected wells are classified as the higher-ranking ‘basic’ or ‘limited’ rungs (WHO/UNICEF 2019). Similarly, having piped water on premises (within the dwelling, yard or plot) is a precondition for a source being classified as ‘safely managed’ (World Health Organization 2017), the highest rung on the ladder. Thus, although the classification has recently been revised to incorporate kiosks (WHO/UNICEF 2018), the classification ambiguities identified through our study could contribute to uncertainty in the indicator used for international monitoring of progress towards SDG Target 6.1.

Our study found comparatively low inter-observer agreement in recording sanitary risk scores, which has several implications for practice and interpretation of previous studies. Several studies have reported no or weak correlation between sanitary risk scores and faecal indicator bacteria counts in source water samples (Ercumen et al. 2017; Luby et al. 2008; Misati et al. 2017). There are several explanations for this, including cross-sectional testing of water quality at a single time point, which may miss transient contamination events, and the use of aggregate scores derived from equally weighted checklist items. Low reliability of sanitary risk observations could also account for such findings. Where sources are prioritised for remediation based on risk scores (Cronin et al. 2006), low score reliability could undermine the objective basis for such decisions. The most experienced observer consistently identified more hazards than the other observers, reflecting similar differences between more experienced, educated staff and colleagues taking measurements in other fields such as anthropometry (Vegelin et al. 2003). To address this issue in large-scale water source surveys incorporating sanitary risk inspection such as the RADWQ surveys, building on current team training recommendations (World Health Organization and UNICEF 2012), it would be possible to include an initial standardisation phase and subsequent follow-up spot-checks on hazard observations. Such an initial phase could test for systematic bias between an experienced observer and other team members, analogously to large-scale anthropometric studies, in which measurers generally undertake an initial standardisation phase, with acceptability metrics such as the Zerfas criteria used to assess observers’ measurement reliability (Li et al. 2016). In routine rural water safety management, such an exercise would likely be logistically challenging however.

There would also be scope to refine current observation checklist items on sanitary risk inspection forms, so as to exclude or refine those with poor inter-observer agreement (Figs. 2 and 3; Online Resource 2). This would require our study protocol to be implemented with a larger number of observers and for a larger and more varied set of water sources. Nonetheless, our study findings suggest that for rainwater systems, roof catchment hazards appeared particularly difficult to assess consistently. Around all water source types, there was seldom significant inter-observer agreement concerning hazards present in the wider environment, such as signs of open defecation or discarded refuse. The items with the strongest inter-observer agreement often related to protection measures at the source itself, such as presence of drainage channels around wells and presence of filter boxes at rainwater tank inlets. This suggests source protection measures are more straightforward to observe consistently than hazards in the wider environment such as garbage or open defecation.

It is possible that the most experienced observer was able to identify hazards by means that were not part of the formal structures of the observation protocol, such as asking questions of source users or bystanders or perhaps simply by experience gained over time in practice. A follow-up naturalistic observation study could help identify such practices and thereby inform protocol refinements. Focussing specifically on livestock and wildlife-related hazards, depending on the item concerned, inter-observer agreement ranged from non-significant to weak. This may lead to exposure misclassification and thereby under-estimation of the contribution of livestock to faecal contamination of water through observational studies of livestock-water contact.

Our findings from rural Kenya indicate much lower agreement between observers recording contamination hazards than we identified in our earlier study in Greater Accra, Ghana (Yentumi et al. 2018). There are several reasons why this might be the case. In this study, the observers came from more diverse backgrounds than in the Ghanaian study. A more diverse set of source types was surveyed in this study, with potential for source type misclassification and consequent use of differing sanitary risk inspection protocols. In the Ghanaian study, the two observers sequentially observed hazards in turn but in one another’s presence, whereas in Kenya, each observer recorded hazards alone. Whilst joint site visits were considered necessary to avoid the risks to the Ghana survey team of lone working, it provided greater opportunity for conferring between observers or one observer’s inspection activities to otherwise influence that of the second observer. It is also possible that the time lag between successive visits by observers in Kenya could have reduced inter-observer agreement. This could have occurred because of environmental changes in the intervening period (e.g. rainfall or source use leading to ponding of water at the source) or if communities sought to remediate perceived hazards following the first observer’s visit. However, we found no correlation between the lag between observer visits and differences in sanitary risk scores.

Our study findings are subject to several further limitations. The survey team may have been subject to a Hawthorne effect, whereby their hazard observations were more rigorous given their participation in this study, though the magnitude of such effects is often unpredictable (McCambridge et al. 2014). Particularly during the first visit, there were some study protocol deviations, with some sources not being visited by all observers, some observers being more likely to visit a source first than others and longer lag times between successive observer visits. However, there was no evidence that lags or being first to visit a source affected hazard score differences between observers. One observer additionally tested water for turbidity and electro-conductivity, and these additional tasks could have affected the hazard observations he made.

Our findings may be difficult to generalise to routine rural water safety management for several reasons. In this study, observers D and F, chosen to typify water user committee members, worked alongside an experienced survey team rather than operating alone, so were less isolated than typical community supply managers. In our study, source types and hazards were recorded via a cell phone application and observers undertook 4 days’ initial training, resources typically unavailable to community supply managers. Since inter-observer agreement deteriorated towards the end of our study, there was however some evidence that survey team fatigue could have contributed to lower agreement in our more prolonged Kenyan study. The more complex design of the Kenyan study reported here could also have resulted in mislabelling of unique identifiers for sources, thereby exacerbating disagreement in the source type classifications and sanitary risk scores recorded by different observers. However, source identifiers were cross-checked by a field supervisor throughout the study.

We have developed a protocol for assessing inter-observer agreement concerning inspection of contamination hazards at six types of water source. Such a study design could be adapted to cover not only risk inspection protocols for other source types and household stored water, but also other field protocols relating to water supplies, most notably field assessments of water point functionality (Bonsor et al. 2018).

## Conclusions

Building on preliminary work in Ghana, we have undertaken the first assessment of inter-observer agreement in water source classification and contamination hazard identification at such sources. Sanitary risk inspection protocols are an appealing tool to support rural water safety management, given their simplicity and the limited resources required to implement such inspections. However, our findings suggest that less experienced observers may miss contamination hazards and inter-observer agreement for some observation checklist items is low. Some hazards were particularly difficult to observe, such as open defecation in the environment around water sources and rainwater harvesting catchment hazards. This suggests that current observation checklists require refinement to address these issues, for example by revising or excluding such checklist items with consistently low inter-observer agreement, avoiding observation of ephemeral hazards, but retaining observation of source protection measures (e.g. concrete aprons and drainage channels around wells).

The independent use by multiple observers of the source type classification used to support international monitoring highlights areas of ambiguity when classifying rural drinking-water sources. In particular, groundwater sources such as boreholes, protected and unprotected wells were often misclassified, as was the distinction between piped water provided on and off premises. We also found that household strategies to cope with water insecurity often led to uncertainty in classifying the sources they used. Such strategies included adapting sources to make use of both piped and rainwater, relying on neighbours for water and using burst pipes where tariffed piped water was unavailable. Whilst a consistent basis for data collection across countries is essential for SDG international monitoring, uncritical use of such water source typologies for other purposes could fail to capture how households cope with water insecurity. This could potentially mean that households’ own means of coping with water insecurity are under-reported and an opportunity for disseminating the most effective strategies more widely is lost. In large-scale surveys, such uncertainty could potentially be reduced through an initial standardisation phase to ensure consistent water source classification use across field team members.

## Electronic supplementary material

Below is the link to the electronic supplementary material.

Supplementary file1 (PDF 285 kb)

Supplementary file2 (PDF 309 kb)

Supplementary file3 (PDF 270 kb)

## Data Availability

The datasets on water sources collected and used during the current study are available from the corresponding author on reasonable request, and are available in the UK Data Archive repository at 10.5255/UKDA-SN-853860. The datasets on precipitation used and analysed in this study are available from the CHIRPS website at http://chg.geog.ucsb.edu/data/chirps/.

## Abbreviations

CHIRPS: Climate hazards group infra-red precipitation with station data
CI: Confidence interval
DHS: Demographic and health survey
JMP: Joint monitoring programme
RADWQ: Rapid assessment of drinking-water quality
SDG: Sustainable development goal
WHO: World Health Organization
