# Supplementary material for: An Assessment of Inter-Observer Agreement in Water Source Classification and Sanitary Risk Observations
Source: Expo Health. 2019 Dec 24;12(4):809–22. doi: 10.1007/s12403-019-00339-3 (PMC7661424; doi:10.1007/s12403-019-00339-3)
Supplement: Supplementary file 1 — Supplementary file1 (PDF 285 kb) [file 12403_2019_339_MOESM1_ESM.pdf]

Article Title: An assessment of inter-observer agreement in water source classification and sanitary risk observations

Journal: Exposure and Health

Joseph Okotto-Okotto[a,]*, Peggy Wanza[b], Emmah Kwoba[b], Weiyu Yu[c], Mawuli Dzodzomenyo[d], SM Thumbi[b,e], Diogo Gomes da Silva[f], Jim A. Wright[c,]*
a Victoria Institute for Research on Environment and Development (VIRED) International, P.O. Box 6423-40103, off Nairobi Road, Rabour, Kisumu, Kenya
b Centre for Global Health Research, Kenya Medical Research Institute, P.O. Box 1578-40100, Kisumu, Kenya
c School of Geography and Environmental Science, University of Southampton, Building 44, Highfield, Southampton SO17 1BJ, UK
d Ghana School of Public Health, University of Ghana, P.O. Box LG 13, Legon, Accra, Ghana
e Paul G Allen School for Global Animal Health, Washington State University, Pullman, WA 99164- 7090, United States of America
f School of Environment and Technology, University of Brighton, Cockcroft Building, Lewes Road, Brighton BN2 4GJ, UK
* Corresponding authors: Joseph Okotto-Okotto: jokotto@hotmail.com Jim A. Wright: j.a.wright@soton.ac.uk

	Piped to premises	Stand pipe	Bore hole	Protected well	Unprotected well	Unprotected spring	Rainwater	Surface water	Water kiosk	Mixed rainwater & piped	Burst pipe	Unprotected spring feeding water pan	Total
Piped to premises	66	2	0	0	0	0	0	0	0	0	0	0	68
Standpipe	3	17	0	0	0	0	0	0	0	0	0	0	20
Borehole	5	0	9	2	0	0	0	0	0	0	0	0	16
Protected well	0	0	3	61	2	0	0	1	0	1	0	0	68
Unprotected well	0	0	0	0	0	0	0	0	0	0	0	0	0
Unprotected spring	0	0	0	0	0	3	0	0	0	0	0	1	4
Rainwater	0	0	0	0	0	0	171	0	0	5	0	0	176
Surface water	0	0	0	1	0	0	0	123	0	0	0	0	124
Water kiosk	1	10	0	0	0	0	0	0	17	0	0	0	28
Mixed rainwater & piped	9	1	0	0	0	0	5	0	0	32	0	0	47
Burst pipe	0	0	0	0	0	0	0	0	0	0	8	0	8
Unprotected spring feeding water pan	0	0	0	0	6	12	0	2	0	0	0	0	20
Total	84	30	12	64	8	15	176	126	17	38	8	1	579
% agreement with experienced observer	78.57%	56.67%	75.00%	95.31%	0.00%	20.00%	97.16%	97.62%	100.00%	84.21%	100.00%	0.00%	

Online Resource 1: Cross-tabulation of water source type classification made by the most experienced observer (JOO - rows) versus four less experienced observers (columns) for 146 water sources during the second visit.
