# Supplementary material for: An Assessment of Inter-Observer Agreement in Water Source Classification and Sanitary Risk Observations
Source: Expo Health. 2019 Dec 24;12(4):809–22. doi: 10.1007/s12403-019-00339-3 (PMC7661424; doi:10.1007/s12403-019-00339-3)
Supplement: Supplementary file 2 — Supplementary file2 (PDF 309 kb) [file 12403_2019_339_MOESM2_ESM.pdf]

Article Title: An assessment of inter-observer agreement in water source classification and sanitary risk observations

Journal: Exposure and Health

Joseph Okotto-Okotto[a,*], Peggy Wanza[b], Emmah Kwoba[b], Weiyu Yu[c], Mawuli Dzodzomenyo[d], SM Thumbi[b,e], Diogo Gomes da Silva[f], Jim A. Wright[c,*]

a Victoria Institute for Research on Environment and Development (VIRED) International, P.O. Box 6423-40103, off Nairobi Road, Rabour, Kisumu, Kenya

b Centre for Global Health Research, Kenya Medical Research Institute, P.O. Box 1578-40100, Kisumu, Kenya

c School of Geography and Environmental Science, University of Southampton, Building 44, Highfield, Southampton SO17 1BJ, UK

d Ghana School of Public Health, University of Ghana, P.O. Box LG 13, Legon, Accra, Ghana

e Paul G Allen School for Global Animal Health, Washington State University, Pullman, WA 99164-7090, United States of America

f School of Environment and Technology, University of Brighton, Cockcroft Building, Lewes Road, Brighton BN2 4GJ, UK

* Corresponding authors: Joseph Okotto-Okotto: jokotto@hotmail.com Jim A. Wright: j.a.wright@soton.ac.uk

**Observation**	**Kappa**	**Z value**	**P value**	**Agreement**
Rainwater **(77 sets of rainwater tank visits)**				
Are there bird droppings on the roof catchment area?	-0.010	-0.26	0.604	None
Are there leaves / plants on the roof catchment area?	0.006	0.16	0.438	None
Are there branches overhanging the roof catchment area?	0.037	0.99	0.162	None
Are there other contamination sources around the roof catchment area?	0.044	1.12	0.132	None
Are the guttering channels that collect water dirty or filled with debris such as leaves?	0.085	1.54	0.062	None
Is there a moveable inlet pipe from the gutter to the tank?	0.023	0.58	0.281	None
Is there a filter box or a sieve at the tank inlet?	0.663	10.42	<0.001	Moderate
Is there debris in the filter box?	0.282	6.31	<0.001	Minimal
Is there any other point of entry to the tank that is not properly covered?	0.175	4.68	<0.001	Minimal
Is there any defect in the walls or top of the tank (e.g. cracks) that could let water in?	0.093	2.52	0.006	Minimal
Is there a depression on top of the tank that would allow ponding?	0.065	1.76	0.039	Minimal
Is there a concrete floor under the tap or place where water is collected?	0.342	8.73	<0.001	Minimal
Is the concrete floor cracked?	0.195	4.83	<0.001	Minimal
Is the concrete floor dirty?	0.317	8.53	<0.001	Minimal

Observation	Kappa	Z value	P value	Agreement
Is the concrete floor broken?	0.130	3.21	<0.001	Minimal
Is the bucket used to collect water left on the ground	0.578	6.34	<0.001	Weak
Does the bucket used to collect water look dirty?	0.497	5.31	<0.001	Weak
Is the water collection area inadequately drained?	-0.111	-3.05	0.999	Minimal disagreement
**Surface water (53 sets of source visits)**				
Is there human habitation visible upstream or uphill of where people collect water?	0.089	2.18	0.015	Minimal
Are any latrines visible upstream or uphill of where people collect water?	0.255	6.28	<0.001	Minimal
Are there any places where garbage is lying upstream or uphill of where people collect water?	0.021	0.52	0.303	None
Are there any cemeteries upstream or uphill of where people collect water?	0.009	0.21	0.418	None
Are there any farm animals kept upstream or uphill of where people fetch water?	0.065	1.60	0.055	None
Are there signs of animals within 3m of the water collection point?	0.015	0.36	0.358	None
Is there a wall or fencing to keep animals out?	N/C			All unfenced.
Is there crop production upstream or uphill of where people fetch water?	0.371	9.05	<0.001	Minimal
**Protected wells (26 sets of source visits)**				
Is there a latrine within 30m of the well?	0.775	12.15	<0.001	Moderate
Is the nearest latrine on higher ground than the well?	0.374	5.91	<0.001	Minimal
Is there any animal excreta in the vicinity of the well?	0.285	4.49	<0.001	Minimal
Is there any human excreta in the vicinity of the well?	0.021	0.31	0.377	None
Is there any garbage in the vicinity of the well?	0.066	1.03	0.150	None
Are there any cemeteries in the vicinity of the well?	0.049	0.57	0.285	None
Are there any animal slaughter areas in the vicinity of the well?	n/c	n/c	n/c	Absent in all cases
Is there any other contamination source in the vicinity of the well?	0.138	2.18	0.015	None
Is the drainage poor, causing stagnant water on the cement floor?	0.343	5.42	<0.001	Minimal
Is there a drainage channel for this well?	1.00	3.79	<0.001	Perfect
Is the drainage channel broken, permitting ponding?	0.282	4.44	<0.001	Minimal
Is there a wall or fencing to keep animals out?	0.164	2.46	0.007	Minimal
Are there signs of animals within 3m of the water collection point?	0.3167	4.98	<0.001	Minimal
Is there a concrete floor around the well?	0.107	1.55	0.061	None
Is the concrete floor less than 1m wide?	-0.030	-0.47	0.680	None

Observation	Kappa	Z value	P value	Agreement
Is there any ponding on the concrete floor?	0.337	5.28	<0.001	Minimal
Is the concrete cracked?	0.262	4.10	<0.001	Minimal
Is the handpump loose at base?	0.035	0.55	0.291	None
Was the cover of the well in place and does it fit?	0.232	3.65	<0.001	Minimal
**Boreholes (9 sets of source visits)**				
Is there a latrine within 30m of the borehole?	0.204	1.58	0.058	Minimal
Is the nearest latrine on higher ground than the borehole?	0.146	1.13	0.130	None
Is there any animal excreta in the vicinity of the borehole?	0.258	1.99	0.023	Minimal
Is there any human excreta in the vicinity of the borehole?	-0.016	-0.10	0.539	None
Is there any garbage in the vicinity of the borehole?	-0.118	-0.90	0.1857	None
Is there any cemeteries in the vicinity of the borehole?	0.0925	0.39	0.349	None
Is there any other contamination source in the vicinity of the borehole?	-0.106	-0.81	0.791	None
Is the drainage poor, causing stagnant water on the cement floor?	0.146	1.13	0.123	None
Is there a drainage channel for this well?	0.414	3.17	<0.001	Weak
Is the drainage channel broken, permitting ponding?	0.1519	0.96	0.1689	None
Is there a wall or fencing to keep animals out?	0.274	2.04	0.021	Weak
Are there signs of animals within 3m of the water collection point?	0.016	0.12	0.4517	None
Is there a concrete floor around the well?	0.654	4.99	<0.001	Moderate
Is the concrete floor less than 1m wide?	0.146	1.05	0.147	None
Is there any ponding on the concrete floor?	0.157	0.84	0.120	None
Is the concrete cracked?	0.1772	1.07	0.1425	None
Is the handpump loose at base?	0.032	0.22	0.411	None

n/c: not calculable.

Online Resource 2: kappa index of agreement statistics for individual hazard observations made independently by six observers in Siaya County, Kenya
