# Supplementary material for: An Assessment of Inter-Observer Agreement in Water Source Classification and Sanitary Risk Observations
Source: Expo Health. 2019 Dec 24;12(4):809–22. doi: 10.1007/s12403-019-00339-3 (PMC7661424; doi:10.1007/s12403-019-00339-3)
Supplement: Supplementary file 3 — Supplementary file3 (PDF 270 kb) [file 12403_2019_339_MOESM3_ESM.pdf]

Article Title: An assessment of inter-observer agreement in water source classification and sanitary risk observations

Journal: Exposure and Health

Joseph Okotto-Okotto[a,*], Peggy Wanza[b], Emmah Kwoba[b], Weiyu Yu[c], Mawuli Dzodzomenyo[d], SM Thumbi[b,e], Diogo Gomes da Silva[f], Jim A. Wright[c,*]
a Victoria Institute for Research on Environment and Development (VIRED) International, P.O. Box 6423-40103, off Nairobi Road, Rabour, Kisumu, Kenya
b Centre for Global Health Research, Kenya Medical Research Institute, P.O. Box 1578-40100, Kisumu, Kenya
c School of Geography and Environmental Science, University of Southampton, Building 44, Highfield, Southampton SO17 1BJ, UK
d Ghana School of Public Health, University of Ghana, P.O. Box LG 13, Legon, Accra, Ghana
e Paul G Allen School for Global Animal Health, Washington State University, Pullman, WA 99164- 7090, United States of America
f School of Environment and Technology, University of Brighton, Cockcroft Building, Lewes Road, Brighton BN2 4GJ, UK
* Corresponding authors: J. Okotto-Okotto: jokotto@hotmail.com J. Wright: j.a.wright@soton.ac.uk

**Observation characteristic**	**unadjusted coefficient (95% c.i)**	**adjusted coefficient (95% c.i)**
Protocol deviations:		
absolute lag between 2 observers' source visits (days)	0.05 (-0.01 to 0.20)	
One of the pair of observers visited the source first	1.49 (-1.50 to 4.49)	
indicators of observer fatigue		
survey took place after 3.30pm	-0.77 (-4.65 to 3.11)	
survey took place at weekend	2.90 (-0.54 to 6.34)	
sequential order of source visits (1=first source visited by observer, etc)	0.06 (0.04 to 0.07)	0.03 (0.02 to 0.05)
Observer (reference category for unadjusted model: Observer B)		
Observer C	-5.38 (-8.51 to -2.25)	-4.88 (-7.12 to -2.60)
Observer D	-0.32 (-4.52 to 3.88)	
Observer E	-0.22 (-5.26 to 8.92)	
Observer F	5.53 (2.48 to 8.58)	5.71 (3.46 to 7.96)
Source type (reference category for unadjusted model: borehole)		
lake / dam	-4.58 (-9.46 to 0.30)	
protected well	-1.59 (-6.69 to 3.50)	
rainwater	9.23 (4.46 to 14.01)	10.70 (8.77 to 12.63)
river/stream	-0.70 (-6.41 to 5.01)	
unprotected spring	-5.72 (-17.52 to 6.08)	
unprotected well	-0.12 (-8.75 to 8.51)	
Environmental change		
absolute difference in rainfall (mm) between days when 2 source visits took place	0.55 (-0.16 to 1.26)	

Online Resource 3: unadjusted (univariate) and adjusted (multivariate) coefficients for a regression model predicting the absolute difference in sanitary risk scores for Observer A versus five other observers
